# The Prospects of *Lactobacillus oris* as a Potential Probiotic With Cholesterol-Reducing Property From Mother's Milk

**DOI:** 10.3389/fnut.2021.619506

**Published:** 2021-03-04

**Authors:** Sadia Afrin, Suraiya Akter, Shamima Begum, Md Nur Hossain

**Affiliations:** ^1^Institute of Food Science and Technology, Bangladesh Council of Scientific and Industrial Research, Dhaka, Bangladesh; ^2^Department of Microbiology, Jagannath University, Dhaka, Bangladesh

**Keywords:** *L. oris*, BioLog^TM^ identification, probiotic, breast milk, cholesterol-lowering

## Abstract

This experiment was conducted to characterize potential *Lactobacillus* spp. isolated from mother's milk and infant feces to obtain new and specific probiotic strains. In this study, seven ascendant strains were identified as *Lactobacillus* spp. based on their morphological characteristics and biochemical properties. Among them, only one (C-1) isolate was identified as *Lactobacillus oris* through BioLog^TM^ identification. The study further investigated the isolate through probiotic potentiality tests such as pH and bile tolerance, NaCl tolerance test, gastric juice tolerance, antioxidant activity, resistance to hydrogen, reduction of sodium nitrate, antimicrobial activity, and antibiotic susceptibility test. The result showed that the strain is a potential probiotic based on probiotic capability. The identified strain was most acid-tolerant and retained around 80% viability for up to 4 h at pH 1.0 and 2.0. The isolate showed tolerance against up to 1.50% bile concentration and gastric juice and was able to grow 1–6% NaCl concentrations. *Lactobacillus oris* showed resistance to most antibiotics as well as antagonistic activity against the tested pathogen, good antioxidant properties, reduction of sodium nitrate and H_2_O_2_. The isolate exhibited good intestinal epithelial adhesion properties, and SDS page was performed for secreted protein analysis. Moreover, the strain showed promising cholesterol-lowering properties based on the cholesterol level. This present result indicates that *L. oris* has superior probiotic properties and can be regarded as a potential probiotic candidate.

## Introduction

Probiotics are alive, non-pathogenic microorganisms (bacteria or yeasts), which when administered in adequate amounts, reach the intestines in sufficient numbers to confer health benefits to the host (1). When selecting probiotics, a number of criteria have to be met by the probiotic organisms, for instance the phenotype and genotype stability, protein and carbohydrate utilization patterns, acid and bile tolerance, intestinal epithelial adhesion properties, production of antimicrobial substances, ability to inhibit pathogens, antibiotic resistance capability, and immunogenicity. Since adhesion to the intestinal mucosa is considered to be a prerequisite for colonization, the ability to adhere to intestinal mucosa is one of the additional importantcriteria for probiotics (2, 3). Before being employed as probiotics, microorganisms must have been granted Generally Regarded as Safe (GRAS) status. In addition, they must have a number of good technological properties such as simple propagation, incorporation into foods, long-term survival, safety in food products, and have clinically validated health effects (1, 4). The majority of probiotics are lactic acid bacteria such as *Lactobacillus* and *Bifidobacterium* species that are part of human and animal intestinal flora (5, 6). In the previous study, a range of lactic acid bacteria were isolated from different sources, especially fermented food products (7, 8). *Lactobacillus* species from Mother's milk deserve significant attention, however, to date, very little information has been reported about probiotics from human breast milk (9–11).

Mother's milk is a vital nutritional factor for the initiation, development, and composition of a child's neonatal gut microbiota. Human breast milk is comprised of a high level of compulsory nutrients for infants and has a valuable effect on the intestinal immune system (12–14). Moreover, it plays a vital role in supporting the survival and development of infants not only for nutrient supply but also for the transfer of microflora originated in breast milk (11). Mother's milk is considered the main source of bacteria. Various studies have reported a different number of live bacteria in mother's milk ranging up to 1 × 10^7^ CFU/mL. The infant takes approximately 300–800 mL of milk per day and thus receives a large number of bacteria within this time (9, 10). The gastrointestinal (GI) microbiota of formula-fed and breastfed infants is considerably different, signifying the importance of the mother's milk microbiota. In recent years, above 200 different species have been expressed in human milk (15). Martin *et al*. isolated *L. gasseri, L. rhamnosus* and *L. fermentum* from breast milk, which have considerable probiotic capabilities (9, 10). A high amount of LAB in milk from a healthy mother plays an essential biological role during the first month of life (10, 16). Studies on this biological fluid specify that human milk is a promising source of potential probiotic bacteria. If these bacteria can protect the host from harmful organisms, they could be attractive probiotic candidates. Recently, research has focused on identifying new strains of *Lactobacillus* with health-promoting properties. Hence, the present study aimed to isolate and identify potential *Lactobacillus* sp., with cholesterol-reducing properties from mother's milk and infant feces for the development of effective probiotics.

## Materials and Methods

### Collection of Milk and Infant Feces Samples

Maternal supplementation with different probiotics may result in an increased occurrence of administered probiotics in breast milk. The infant consumes breast milk directly so the same microbiota can be found in infant feces. Based upon this rationale, mother's milk and infant's feces may be a potential natural source to isolate effective strains of *Lactobacillus* sp. In the present study, mother's milk and infant facial samples were collected from Dhaka Medical College Hospital, Bangladesh. Fifteen (15) healthy mothers and infants were targeted for a period of 1–5 days after birth, between the ages of 24–35 years, and the feces samples were taken from neonatal within the age range from 2 to 5 days (Table 1). We collected neonatal samples within this timeframe because fecal microbiota undergoes progressive succession before the maturation period of 1–5 days (17). The microbiological analysis were performed in triplicate. The studies concerning human participants were reviewed and approved by the BCSIR Institutional Ethical Review Committee. The participants or their legal guardians (sample donors) provided written consent to take part in this study. Samples were taken and collected in sterile tubes and immediately transported to the Industrial Microbiology Laboratory, IFST, BCSIR, Dhaka. It is necessary to mention that all the sample donors were healthy, using a normal diet, and did not take antibiotic treatments during pregnancy or after birth.

**Table 1:** Samples collected from mothers and their infants.

**Sample ID**	**Sample type**	**Age (Mother and Infant)**
S-1	M	25 years & 2 days
S-2	M	28 years & 3 days
S-3	M	23 years & 5 days
S-4	F	20 years & 3 days
S-5	F	28 years & 3 days
S-6	M	27 years & 2 days
S-7	F	27 years & 5 days
S-8	M	21 years & 3 days
S-9	M	30 years & 2 days
S-10	F	19 years & 2 days
S-11	F	25 years & 3 days
S-12	F	24 years & 3 days
S-13	F	34 years & 2 days
S-14	M	25 years & 2 days
S-15	M	35 years & 3 days

*M, Milk; F, Feces*.

### Chemicals and Reagents

SDS-polyacrylamide gel (Invitrogen, Germany), Coomassie Brilliant Blue (Promega, USA), Biochemical reagent, Microbiological media, antibiotic disks (Hi-Media, India), and all other chemicals and enzymes used from Sigma, USA.

### Sample Preparation

The samples were preserved at 4°C before and after processing. Approximately, 1 g of fecal material or 1 mL of milk sample was added to 100 mL of buffered peptone water, thus homogenized thoroughly and incubate at 37°C for 30 min. Both of the samples were serially diluted in phosphate buffer (0.1 M, pH 6.2). The sample preparations were executed according to Lackey et al. and Gharbi et al. (18, 19).

### Isolation of Lactic Acid Bacteria

The prepared inoculum was plated on a selective medium up to 10^5^ dilution using pour plate technique to isolate potential *Lactobacillus* species. The plates were incubated anaerobically for 24–48 h at 37°C. Individual colonies were selected and transferred into a sterile MRS broth medium. The preferred colonies were purified with a streak plate technique based on colony morphology, gram staining, catalase, and oxidase reaction as well as different carbohydrate utilization. Based on biochemical properties, only the selected strains were identified through BioLog^TM^.

### BioLog^TM^ Identification System

BioLog^**TM**^ (BIOLOG™, USA) is an advanced microbial identification system that can swiftly identify species of aerobic and anaerobic bacteria, yeasts, and fungi. The latest generation redox chemistry of the BioLog^**TM**^ enables testing and identification of aerobic gram-negative and gram-positive bacteria in the same test panel (20). For the species-level identification of predicted *Lactobacillus* sp. BioLog^TM^ identification system was used based on 23 Chemical Sensitivity Assays with 71 carbon sources in GEN-III microplate. The isolates were cultured on BUA, which is a universal growth media for anaerobic bacteria. The microplates and inoculating fluids were pre-warmed at 37°C for 30 min. After 18 h incubation, the inoculum of bacterial culture was added to the inoculating fluid-A (21). The cell suspension was then poured into the multichannel pipette and all 96 wells were inoculated with exactly 100 μL of the bacterial suspension. The microplate was then incubated anaerobically for 24–48 h at 37°C. All the wells start out colorless when inoculated. During incubation, there is increased respiration in the wells where cells can use a carbon source. Increased respiration causes a reduction of the tetrazolium redox dye, forming a purple color. The negative well (A-1) remained colorless, as it contains no carbon source. There is also a positive control well (A-10) used as a reference for the chemical sensitivity assays. After incubation, the microplate was placed into the Micro Station Reader and the result was given by comparing with the database using the software program MicroLog 4.20.05 (BIOLOG™, USA) (22).

### Probiotic Properties of Isolate

According to the FAO and the WHO, before demonstrating an organism is probiotic, that microorganism must rely on some technological properties such as surviving in acidic situations, persisting even though digestive enzymes occur in the stomach, resistance to bile salts at the commencement of small intestine, and antimicrobial activity as well as antibiotic susceptibility (1, 23).

### Growth at Different Temperatures

Modified MRS broth was prepared and transferred to 5 mL tubes. After that, 50 μL of overnight cultures were inoculated to tubes and incubated anaerobically for 24–48 h at 10, 20, 30, 35, 37, 40, and 45°C. Cell growth was observed and measured by capturing absorbance by a spectrophotometer at 660 nm (Thermo Multiskan EX).

### Viability at Different pH

The acid tolerance of the bacterial isolate was observed by incubating the organisms with different pH in MRS broth. Resistance to pH 3 is often used *in vitro* assays to determine the resistance to stomach pH as foods remain at the stomach for 3 h (24). To determine the growth in diverse pH, 100 μL overnight cultures of the isolate were inoculated into MRS broth, with varying pH ranging from 1.0–7.0, adjusted with 1 M HCl by pH meter (Hanna Instruments, Italy). The inoculated broths were incubated anaerobically for 4 h at 37°C and growth was observed at OD 600 nm.

### Growth at Different Concentration of NaCl

The designated isolate was examined for tolerance against different levels of NaCl concentrations (1.0–7.0%). The inoculated broths were anaerobically incubated at 37°C for 24 h and growth was monitored at OD 600 nm.

### Tolerance to Bile Salt

The growth rate of bacterial culture was determined in MRS broth at pH 3.0 containing different levels of bile salt concentration (0.3, 0.5, 1.0, 1.5, and 2.0%). An inoculum of probiotic bacteria was prepared in MRS broth by overnight incubation and 1% (v/v) fresh culture was inoculated to the tube containing bile salt after sterilization. The broth was incubated anaerobically overnight at 37°C and the growth of the probiotic bacteria was compared with that of MRS broth as control (25). The survival rates of the isolates were dignified by taking absorbance at 600 nm by a spectrophotometer.

### Resistance to Simulated Gastrointestinal Digestion

The solutions for simulated gastrointestinal digestion were organized according to Minekus et al. (26). During gastrointestinal digestion, the survivability of probiotic isolate was evaluated using an *in-vitro* digestion method. The *in vitro* gastrointestinal digestion procedure applied here consisted of a three-step model, which sequentially simulated the digestion in the mouth, stomach, and the small intestine as expressed by Minekus et a. and Gut et al. (26, 27) with few modifications. In this process, simulated Silivary Fluid (SSF) was arranged by dissolving 0.1126 g KCL, 0.0503 g KH_2_PO_4_, 0.1142 g NaHCO_3_, 0.0030 g MgCL_2_(H_2_O)_6_ and 0.0006 g (NH4)_2_CO_3_ in 70 mL Milli-Q water, and finally, pH was adjusted to 7.0. Simulated Gastric Fluid (SGF) was prepared by dissolving 0.0514 g KCL, 0.0122 g KH_2_PO_4_, 0.210 g NaHCO_3_, 0.2761 g NaCl, 0.0024 g MgCL_2_(H_2_O)_6_ and 0.0048 g (NH4)_2_CO_3_ in 70 mL Milli-Q water and adjust pH to 3.0. Simulated Intestinal Fluid (SIF) was organized by dissolving 0.0507 g KCL, 0.0108 g KH_2_PO_4_, 0.714 g NaHCO_3_, 0.2246 g NaCl, and 0.0067 g MgCL_2_(H_2_O)_6_ in a 70 mL Milli-Q water thus adjust pH to 7.0. The samples were incubated anaerobically at 37°C in a shaking incubator and the colonies were counted accordingly. α-amylase from human saliva, porcine pepsin, and porcine pancreatic lipase enzymes were employed for mouth mastication, gastric and intestinal digestion, respectively.

### Hydrophobicity

The cell surface hydrophobicity was assessed with n-hexadecane and chloroform. Cells were suspended in 3 mL of 50 mM potassium phosphate buffer at pH 7.0 (28, 29). The suspensions were centrifuged (3-18KS centrifuge, Sigma, Germany) at 10,000×*g* for 5 min at 4°C. Pellets were collected, washed twice, and then resuspended with the same buffer. Absorbance at 600 nm was measured and considered as A_0_. One milliliter of the suspension was mixed with 200 μL of n-hexadecane and chloroform by vortexing for 120 s. The two phases were allowed to separate for 1 h at room temperature. The lower aqueous layer was cautiously shifted to hygienic tubes and absorbance was determined as A. Changes in the absorbance of probiotic bacterial suspension were recorded at 600 nm by a spectrophotometer.

Surface hydrophobicity (*SHb*%) was determined using the following formula:

$$\begin{array}{l} SHb\%=\frac{\text{A}0-\text{A}}{\text{A}0}\;\times \;100 \end{array}$$

Here, A_0_ and A are the absorbances before and after extraction with chloroform and n-hexadecane, respectively. For statistical analysis, all *SHb* experiments were repeated three times.

### Autoaggregation Ability

The culture was harvested from broth medium by centrifugation (5,000×*g*, 5 min), washed, and resuspended with normal saline solution (28, 30). The absorbance was measured at 0, 2, and 24 h by a spectrophotometer at 600 nm without shaking the cell suspension. The autoaggregation was estimated as follows:

$$\begin{array}{l} \text{Autoaggregation }\left(\%\right)=\left(1-\frac{At}{A0}\right)\times 100 \end{array}$$

A_0_ is the initial absorbance at 600 nm and A_t_ is the absorbance at 600 nm.

### Co-aggregation

The co-aggregation capacity of the *L. oris* probiotic stains with other pathogenic and non-pathogenic bacteria was assessed following the method of Vlková et al. and Keller et al. (31, 32) with some modifications. The colon intestine contains millions of both pathogenic and non-pathogenic bacteria. The *L. oris* along with *L. rhamnosus* LGG, *L. casei* 431, *B. animalis* subsp. lactis 12, *Escherichia coli* ATCC 11303, *Salmonella typhi* ATCC 13311, *Shigella flexneri* ATCC 12022, *Enterobacter faecalis* ATCC 29212, *Vibrio parahemolyticus* ATCC 17802, *Pseudomonas aeruginosa* ATCC 27853, and human fecal culture (collected from Industrial Microbiology lab stock, IFST, BCSIR) were used for the co-aggregation activity. The bacterial cell suspension was prepared with the final density of 9.0 log CFU/mL. The mix of pathogenic bacteria and probiotic cells of 3.0 mL each were dispensed into sterile tubes. The tubes were thoroughly mixed and incubated for 60 min at 37°C. The absorbance was taken at 600 nm by a spectrophotometer. Pathogenic and probiotic bacteria were prepared separately and absorbance was taken as control. The percentage of co-aggregation was calculated according to the formula:

$$\begin{array}{l} \text{Co}-\text{aggregation }\left(\%\right)=\frac{\left(\text{Ax }+\text{ Ay}\right)/2-\text{A}\left(\text{x }+\text{ y}\right)}{\left(\text{Ax }+\text{ Ay}\right)/2}\times 100 \end{array}$$

where Ax–represents the absorbance of the probiotic strain, Ay–represents the absorbance of the pathogenic bacteria under study; A (x + y)–represents the absorbance of the mixture of both.

### Assessment of Antimicrobial Activity

The selected isolate was investigated for antimicrobial activity against a variety of indicator organisms (pathogens) by the modified method of Arques et al. (33). The plates were poured with 20 mL Muller Hinton Agar (MHA) medium. Nine different human pathogens belonging to both gram-positive and gram-negative groups such as *Bacillus cereus* ATCC 10876*, Bacillus subtilis* ATCC 11774, *Staphylococcus aureus* ATCC 9144, *Escherichia coli* ATCC 11303, *Salmonella typhi* ATCC 13311, *Shigella flexneri* ATCC 12022, *Enterobacter faecalis* ATCC 29212, *Vibrio parahemolyticus* ATCC 17802, *Pseudomonas aeruginosa* ATCC 27853 were used in this study. The pathogenic strains were grown in nutrient broth (NB) for 24 h and spread on an MHA agar plate. Three wells (6 mm) of each plate were prepared through a sterile borer and the supernatant of the isolate was placed into the well. The plates were pre-inoculated at room temperature for the diffusion and incubated anaerobically overnight at 37°C and observed for zones of inhibition.

### Antibiotic Susceptibility Test

The selected strain was examined for the antibiotic resistance pattern as recommended by the Clinical and Laboratory Standards Institute (CLSI; Wayne, PA, USA) (34). The isolates were grown overnight in MRS broth at 37°C. Petri dishes containing 20 mL of MRS were allowed to solidify at room temperature and swabbed with organisms. In the present study, 10 different types of antibiotic disks viz ampicillin (10 μg), erythromycin (15 μg), gentamicin (10 μg), kanamycin (30 μg), neomycin (10 μg), vancomycin (30 μg), penicillin G (10 μg), nalidixic acid (30 μg), nitrofurantoin (300 μg) and doxycycline (30 μg) were used. The plates were incubated anaerobically for 24 h at 37°C. The diameter (mm) of the inhibition zone was measured with an antibiotic zone scale. Results were expressed in terms of resistance, moderate susceptibility, or susceptibility by comparing with the zone diameters provided in the performance standards for antimicrobial disc susceptibility tests.

### Antioxidant Activity Determination

To prepare intracellular extracts, the overnight cultured bacterial isolate was centrifuged at 12,000 rpm at 4°C for 3 min. The pellet of the cell was washed twice and re-suspended in water (pre-autoclaved) and ultrasonicated at 30 KHZ for 3 min, with an intermission of 1 min. Subsequently, the cellular debris was removed by centrifugation at 200×*g* at 4°C for 15 min. The cell-free supernatant was used as an intracellular extract to assess the antioxidant activity (3). The antioxidant activity of intact cells and intracellular extracts were determined by the DPPH (2, 2-Diphenyl-1-picrylhydrazyl) free radical scavenging assay method. The control was prepared with PBS buffer at pH 7.4. The absorbance was measured by UV spectrophotometer at 517 nm, the readings were recorded in triplicate and the average absorbance value was calculated. The antioxidant activity of the samples was expressed as the percentage (%) of radical scavenging as follows:

$$\begin{array}{l} \text{DPPH activity }\left(\%\right)=\left[\left(Ac-As\right)/Ac\right]\times 100 \end{array}$$

Where, *Ac*= absorbance of the control and *As* = absorbance of the sample.

### Resistance to Hydrogen Peroxide

Resistance to hydrogen peroxide was determined according to Serata et al. (35). Freshly prepared bacterial culture (10 mL) was supplemented with 0.85% (w/v) NaCl (10 mL), thus added variable concentrations (0.5, 1.0, and 1.5 mM) of H_2_O_2_. The viability of the isolate was resolute after 1 h of incubation.

### Sodium Nitrite Depletion

Sodium nitrite depletion of the selected isolate was carried out according to Wu et al. (36). Newly prepared bacterial culture (100 μL) was inoculated and incubated anaerobically for 48 h at 37°C. Sterilized sodium nitrite (1 mL) was combined with 9 mL of MRS broth. For control samples, sterile water was applied as a substitute for inoculum. The colorimetric nitrite method was employed to measure initial and final absorbance at 538 nm against a blank. The depletion of sodium nitrite was calculated as follows:

$$\begin{array}{l} \text{Nitrite depletion }\left(\text{\%}\right)=\left(1-\text{Ci}/\text{Cf}\right)\times 100 \end{array}$$

Whereas, C_i_ = Amount of nitrite present in MRS broth at 0 h; C_f_ = Amount of nitrite present in MRS broth after 48 h.

### Test for Safety Evaluation

#### Hemolytic Activity

The hemolytic activity was determined according to the method of Yadav et al. (37). The tested isolate was streaked in a blood agar medium and incubated at 37°C for 48 h. The plates were observed for β-hemolysis, α-hemolysis, and non-hemolytic activities after the incubation period.

#### DNase Activity

To test for DNase enzyme production the selected isolates were streaked onto a deoxyribonuclease (DNase) agar medium. Thus, the plates were incubated at 37°C for 48 h and examined for the zone of DNase activity. An understandable zone (pinkish) around the colonies was determined as positive DNase activity (38).

#### Partial Analysis of Bacterial Protein by SDS-PAGE

The isolates were grown in MRS broth at 37°C for 48 h anaerobically. The culture broth was sonicated for cell disruption and centrifuged at 10,000 rpm for 15 min at 4°C. The supernatant was collected and cold acetone (−20°C) was added to the supernatant and mixed vigorously. The supernatant was incubated at −20°C for 1 h and centrifuged at 10,000 rpm for 15 min at 4°C. Cell pellets were collected, disposing of supernatant and allowing the uncapped tube to evaporate acetone completely. After adding 2% SDS, the whole-cell protein extract was stored at 4°C until use (39). Whole-cell protein extract was mixed with an equal volume of sample loading buffer and heated for 10 min at 80°C. The processed samples were then loaded onto 7.5% SDS-polyacrylamide gel wells as illustrated by Paul (40). Electrophoresis was performed at 30 mA until the tracker dye (Bromophenol blue) reached the bottom of the gel. Gels were stained for protein with Coomassie Brilliant Blue (CBB) destaining with a solution containing 6.75% (v/v) glacial acetic acid and 9.45% (v/v) methanol. The molecular weights were determined by SDS-PAGE protein molecular markers.

#### *In vitro* Cholesterol-Lowering Activity

Water-soluble cholesterol (polyoxyethanyl-cholesterylsebacate, Sigma) was used for *in-vitro* cholesterol reduction assay. The *L. oris* was grown in modified MRS broth at 37°C anaerobically. The cholesterol was added to milk to make yogurt (41) with *Lactobacillus delbrueckii* sub sp. *bulgaricus* and *Streptococcus thermophilus* at a final concentration of 25–100 mg/100 mL, inoculated with 2% of *L. oris* (pellets) (42), and incubated at 39°C for 6–7 h. The cholesterol content was assessed by a modified colorimetric method as described by Gilliland et al. (43). The activity of cholesterol-lowering (mg/mL) was calculated as follows:

$$\begin{array}{l} Chlesterol\;lowering\;activity,\;\%=\\ \;\frac{cholesterol\;added-cholesterol\;recovered}{cholesterol\;added}\times 100 \end{array}$$

#### Statistical Analysis

All the experiments were executed in triplicate. The diversity index data were statistically analyzed by one-way analysis of variance (ANOVA). Data were presented as the mean ± standard deviation (SD) for the indicated number of separately completed experiments. Data analyses were finalized by SPSS software (version 20.0; SPSS Inc., Chicago, USA).

## Results

### Isolation of *Lactobacillus* sp.

The properly diluted samples were incubated anaerobically for 48 h at 37°C and the small bacterial colony with entire margin, creamy white shiny color, convex elevation, and opaque opacity were preliminarily screened as *Lactobacillus* spp. The presumed colonies were chosen for further study. To determine the morphological characteristics, the selected strains were examined by an optical microscope (Olympus, Japan). *Lactobacillus* spp. are the genus of gram-positive rod-shaped and non-spore-forming bacteria. The isolates that exhibited similar characters as *Lactobacillus* sp. were selected for further characterization.

### Biochemical Characterization

To recognize the characteristics of *Lactobacillus* sp. some essential biochemical tests were accomplished. The results of biochemical tests were listed in Table 2. The carbohydrate fermentation of the isolate was determined on carbohydrate broth supplemented with 1% (w/v) of the following carbohydrates: arabinose, ribose, lactose, fructose, sucrose, maltose, glucose, rhamnose, galactose, and raffinose.

**Table 2:** Biochemical characterization of potential *Lactobacillus* isolates.

**Result of the biochemical test**							
**Test**	**C-1**	**C-2**	**C-3**	**C-4**	**C-5**	**C-6**	**C-7**
Catalase	–	–	–	+	–	–	+
Oxidase	–	–	–	–	–	–	–
Gas from Glucose	+	+	+	–	+	–	+
Gelatin hydrolysis	–	–	–	+	–	+	–
H_2_S production	–	–	–	–	–	–	–
Indole production	–	–	–	–	–	–	–
Motility	–	–	–	+	–	–	–
Methyl Red	–	–	–	–	–	+	–
VP (Voges)	–	–	–	–	–	+	–
Urease	–	–	–	–	–	+	–
**Result of the fermentation test**							
Arabinose	+	+	+	–	+	–	+
Ribose	+	+	+	+	+	+	+
Lactose	*	*	–	–	*	–	+
Fructose	+	–	+	+	+	–	+
Sucrose	+	+	–	+	–	+	+
Maltose	+	+	–	+	+	–	+
Starch	+	+	+	+	+	+	+
Rhamnose	+	+	+	–	+	+	+
Galactose	+	+	+	+	+	+	+
Raffinose	*	*	*	*	+	*	*

Readings were recorded anaerobically after 24 h at 37°C; Key: +, Positive reaction; –, Negative reaction;

* *, Weak reaction*.

### Identified by Biolog^TM^ System

For species-level identification of the selected bacterial isolate BIOLOG^TM^ system was carried out. This system intends to give a quick, suitable approach to bacteria identification with 3,000 species databases. The result specifies that only one bacterial strain identified as *L. oris* (Supplementary Figure 1). Table 3 illustrates the outcome of the BIOLOG^TM^ identification system. For additional verification, this isolate was examined using three replications. This isolate was finally chosen for detailed observation.

**Table 3:** Microorganism identification with BIOLOG^TM^ system.

**Strain**	**ID**	**PROB**	**SIM**	**DIST**
	**(identification)**	**(probability)**	**(similarity index)**	**(distance)**
C-1	*Lactobacillus oris*	0.582	0.582	6.160

### Assessment of *In-vitro* Probiotic Activities of *Lactobacillus* spp.

#### Growth at Different Temperatures

**Figure 1:**
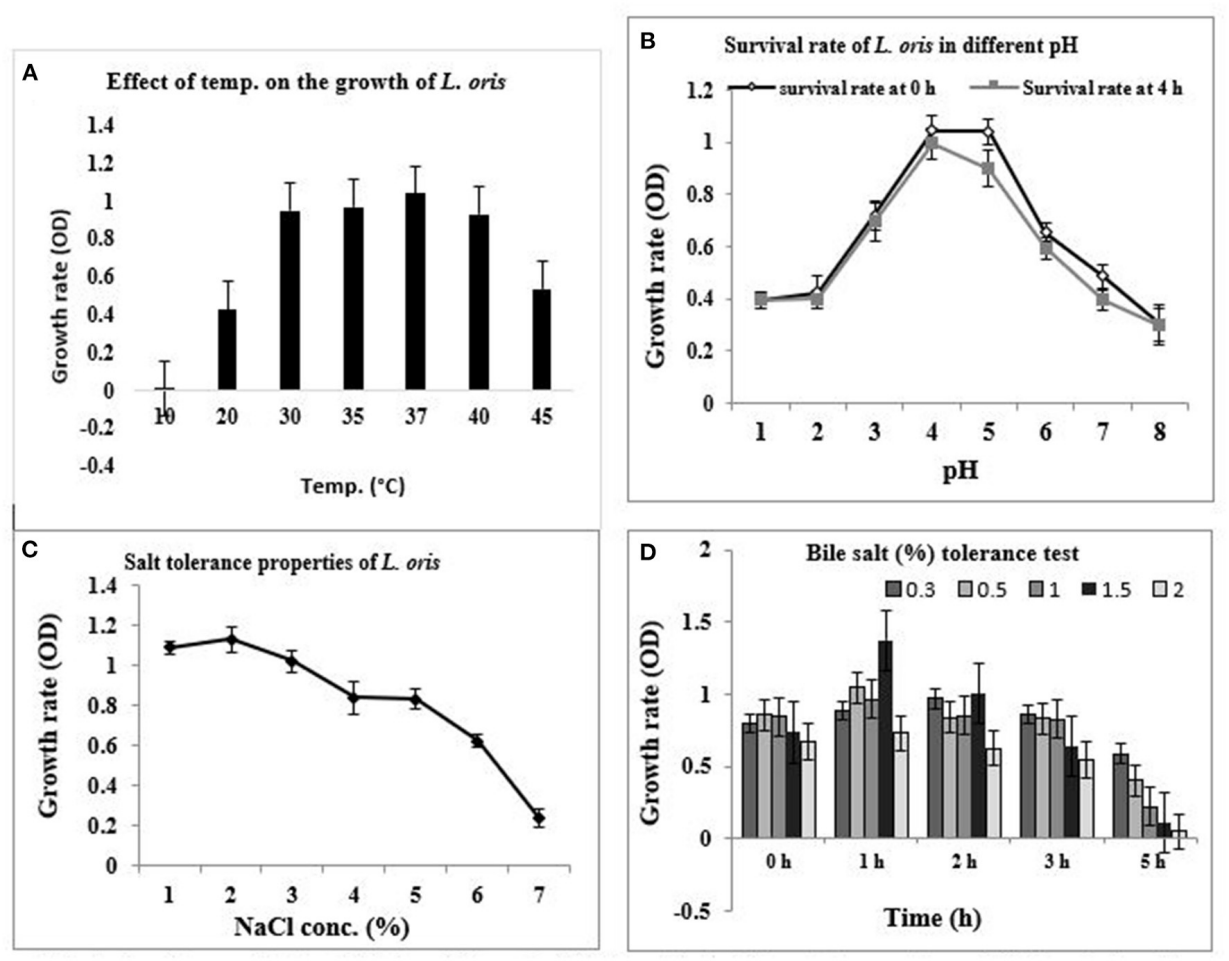
*In-vitro* probiotic attributes of isolated strain *L. oris*. **(A)** Growth rate at different temperatures, **(B)** Survival rate with different pH at 37°C for 0 h and 4 h, **(C)** Effect of NaCl concentration, **(D)** Effect of bile salt (%) concentration under variable periods of time at 37°C.

Since the characterization of probiotics is determined by their capability to survive the upper digestive tract and colonize in the intestinal lumen and colon, the temperature is a vital criterion. Figure 1A shows the growth of *L. oris* at different temperatures. The results indicate that the isolated organism grew best at 37°C, was also able to grow at 45°C, but unable to grow at 10°C.

#### pH Tolerance Assay

The pH condition of the stomach is acidic. The pH tolerance of the isolate was measured by growing the isolate in a varying pH environment. The capacity of the strain to survive in the acidic pH at 0 h and 4 h of incubation at 37°C is presented in Figure 1B. The results revealed that *L. oris* retained around 80% viability for up to 4 h at pH 1 and 2. No considerable differences were obtained between the viability after 4 h, indicating acid resistance-capacity to intestinal pH conditions. The result also indicated that the strain could stay alive up to pH 8.0.

#### Measurement of Salt (NaCl) Tolerance

Sodium chloride tolerance was measured by testing the ability to grow in the presence of various concentrations of NaCl. According to the test results, *L. oris* can grow up to 7.0% NaCl concentration but there was a rapid decrease in survival rate after 5.0% of NaCl concentration (Figure 1C).

#### Measurement of Bile Salt Tolerance

Bile salt tolerance assists in the *in vitro* assessment of metabolic activity and colonization of isolates in the small intestine. The effect of bile salt on the growth and survival rate of the identified isolates is shown in Figure 1D. The bile salt tolerance of *L. oris* was measured by growing isolates in a varying bile salt concentration at 37°C. *L. oris* was found to be the resistant strain, it showed viability at 1.5% of bile concentration after 5 h exposure.

#### Gastric Juice Tolerance

**Figure 2:**
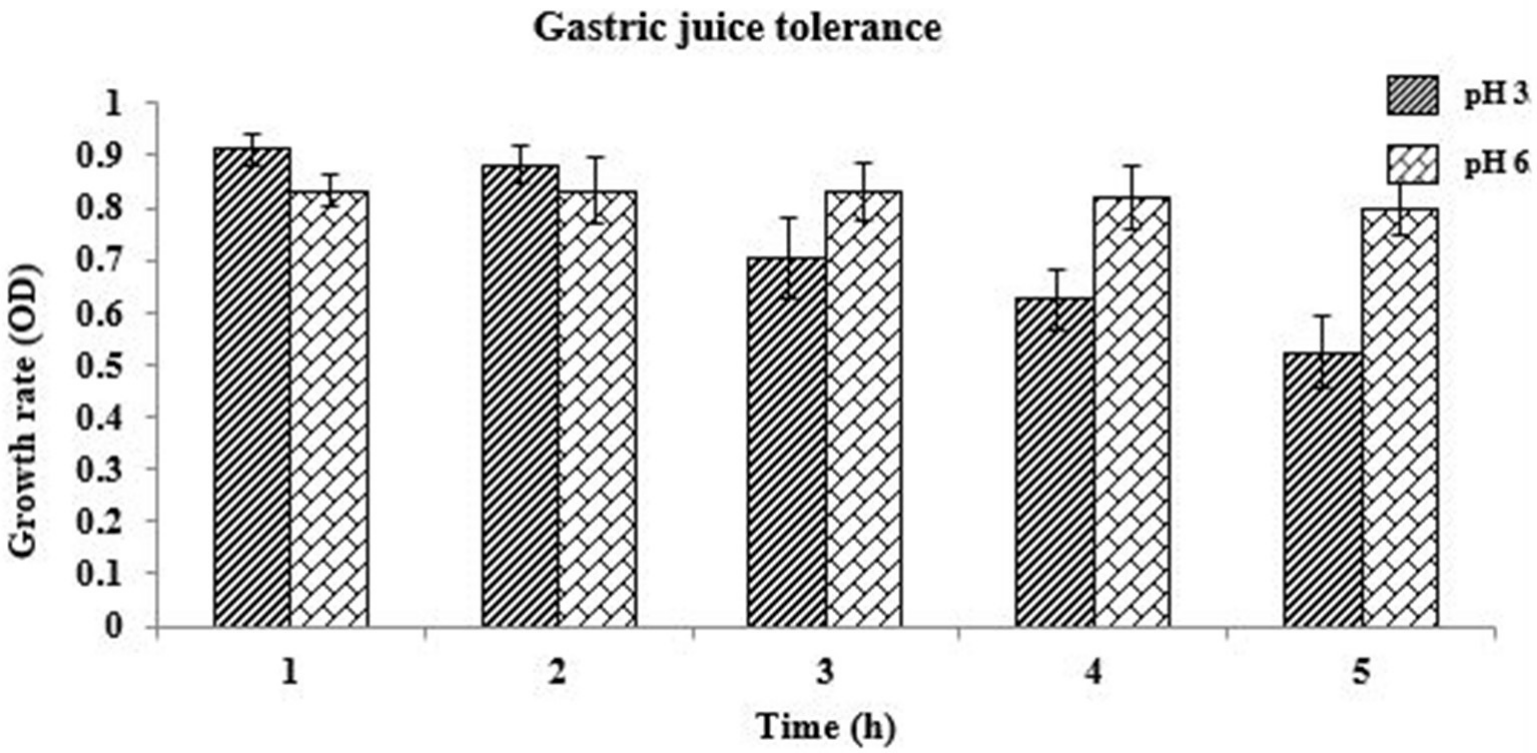
Gastric juice tolerance of *L. oris* at pH 3.0 and 6.0.

The capability to adhere to intestinal surfaces is a vital selection criterion for potent probiotics because adhesion to the intestinal epithelial tissue is considered to be a criterion for colonization and thus producing health benefits by killing the detrimental pathogens and balancing the microbiota balance in the Gastrointestinal Tract. The effect of simulated gastric juice (pH 3.0 and 6.0) on the identified isolate is presented in Figure 2. *L. oris* showed better viability in gastric juice for 3 h at pH 3.0. After 3 h the viability decreased. The viability of the *L. oris* on gastric juice at pH 6.0 was used as a control.

#### Cell Hydrophobicity, Auto, and Co-aggregation Activity

**Figure 3:**
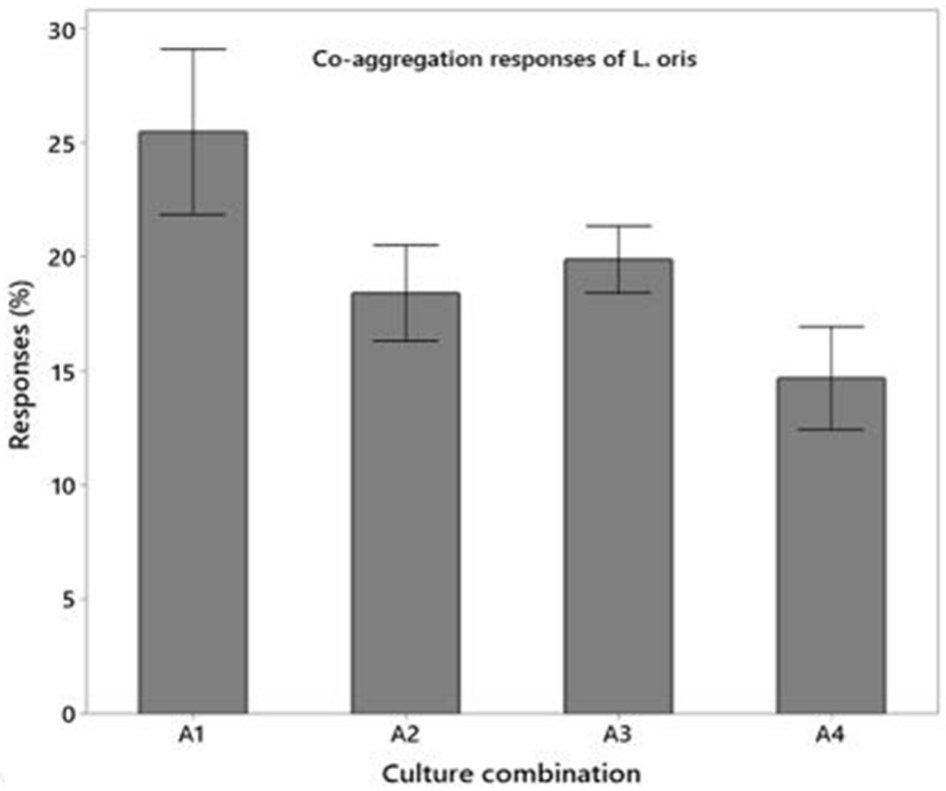
Co-aggregation activities of *L. oris* probiotic with pathogenic and non-pathogenic bacteria.

The hydrophobic properties of *L. oris* displayed a superior affinity to chloroform compare to n-hexadecane that are 91.5 ± 2.43% and 78.32 ± 1.92%, respectively. The autoaggregation capacity of *L. oris* increases over time. The fast autoaggregation obtained showing percentages of 48.93 ± 1.20% at 2 h and 92.75 ± 3.78% at 24 h. Figure 3 demonstrated the co-aggregation activity of the probiotic *L. oris* strain.

#### Antagonistic Activity Measurement

**Figure 4:**
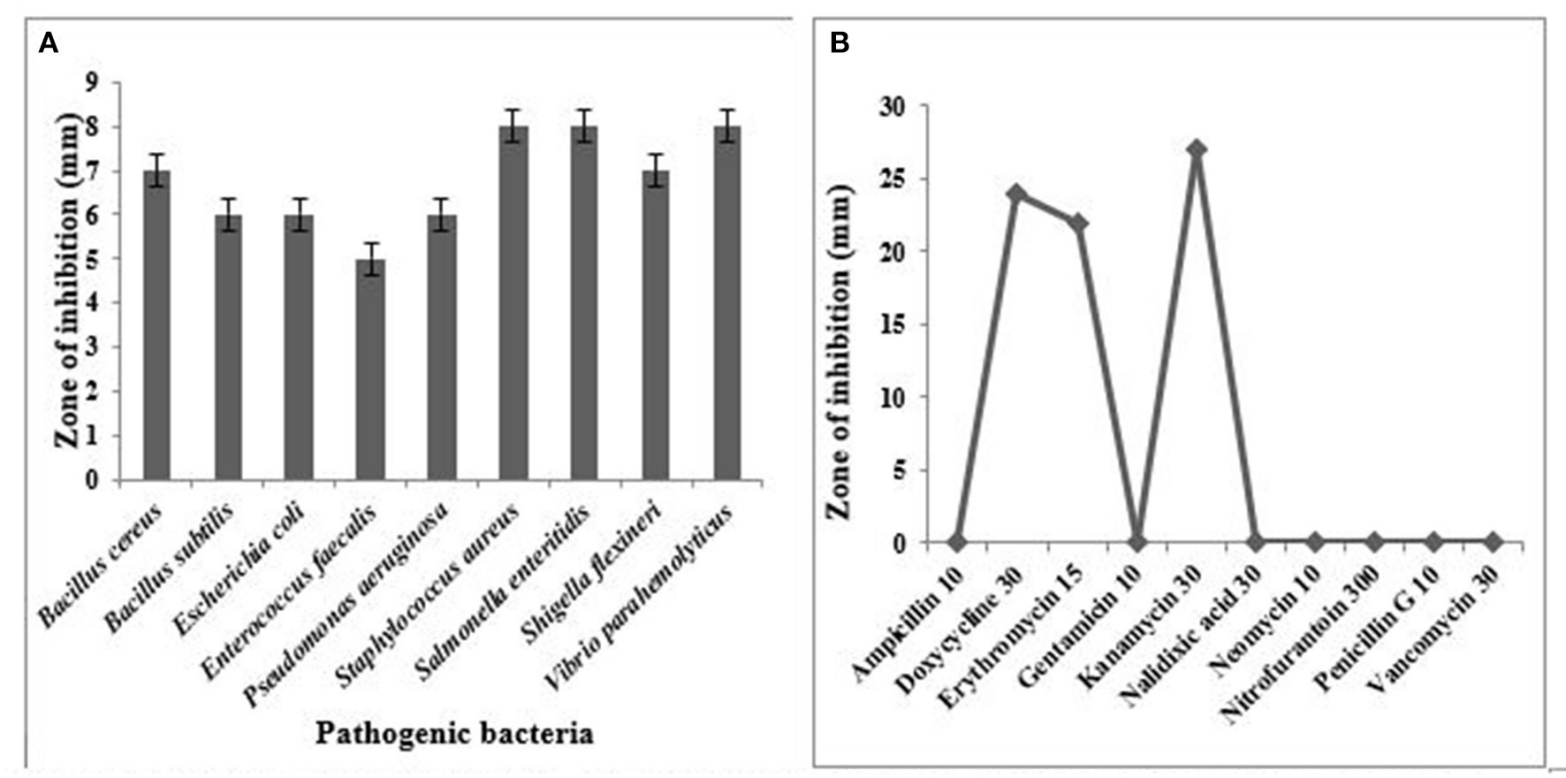
**(A)** Antimicrobial activity of *L. oris* against test pathogens, **(B)** Antibiotic resistance pattern of isolated strain *L. oris*.

It has been well-documented that *Lactobacillus* spp. showed very good antagonistic properties by producing numerous antimicrobial compounds. *L. oris* was observed for their antimicrobial activity against the indicator microorganisms. Nine human pathogens belonging to both gram-positive and gram-negative were employed in this study. The result proved *L. oris* has significant antibacterial activity against all the tested pathogens with significant variations. The *L. oris* exhibited the highest inhibition activity against *V. parahaemolyticus, S. enteritidis*, and *S. aureus* and the lowest inhibition activity against *E. faecalis*. The diameters of zones of inhibition were given in Figure 4A.

### Antibiotic Susceptibility Profile

The *L. oris* was screened for susceptibility against various antibiotics. For appropriate selection generally, two groups of antibiotics are suggested in EFSA guidelines including inhibitors of cell wall synthesis (ampicillin and vancomycin) and inhibitors of protein synthesis (gentamycin, neomycin, erythromycin, kanamycin, and others). The results were compared with the zone size interpretative chart. In the present study, the tested isolate was sensitive to doxycycline, erythromycin, and kanamycin. A variable antibiotic sensitivity was observed and reported in Figure 4B.

### Antioxidant Activity

The antioxidant activity of intact cells and intracellular extracts of *L. oris* was estimated by DPPH free radical scavenging assay. The potential isolate *L. oris* exhibited a higher ability to scavenge the radical DPPH and was determined as 31.14%. This finding agreed with the previous study for *Lactobacillus* isolated from fermented foods (44). The intracellular cell-free extracts of *L. oris* showed 30.32% scavenging ability.

### Resistance to Hydrogen Peroxide

The potential isolate *L. oris* can resist H_2_O_2_. Results exposed a considerable impact on the viability of the isolate were observed at the concentration of H_2_O_2_ increases, exhibiting a decreasing trend. The isolate showed the maximum viable cell count at 0.5 mM of H_2_O_2_ (8.3 logs CFU/mL) while the least was observed at 1.5 mM H_2_O_2_ (5.0 log CFU/mL).

### Depletion of Sodium Nitrite

Sodium nitrite is a salt that is used as an additive in food products especially in meats to keep characteristic color and flavor. Due to safety issues, sodium nitrite is considered a carcinogenic substance for foods. Therefore, controlling their concentrations is important for maintaining a safe food supply. The obtained result revealed that *L. oris* had the ability to deplete nitrite as of 85.0%.

### Safety Evaluation of Isolate

The safety assessment of *L. oris* was decided by their hemolytic and DNAse activities that demonstrated the non-pathogenic category of the probiotic isolate. The results of the current study revealed “no zone” in the test plates inoculated with the isolate, thus confirmed no hemolytic or DNAse activities.

### Partial Analysis of Bacterial Metabolites

**Figure 5:**
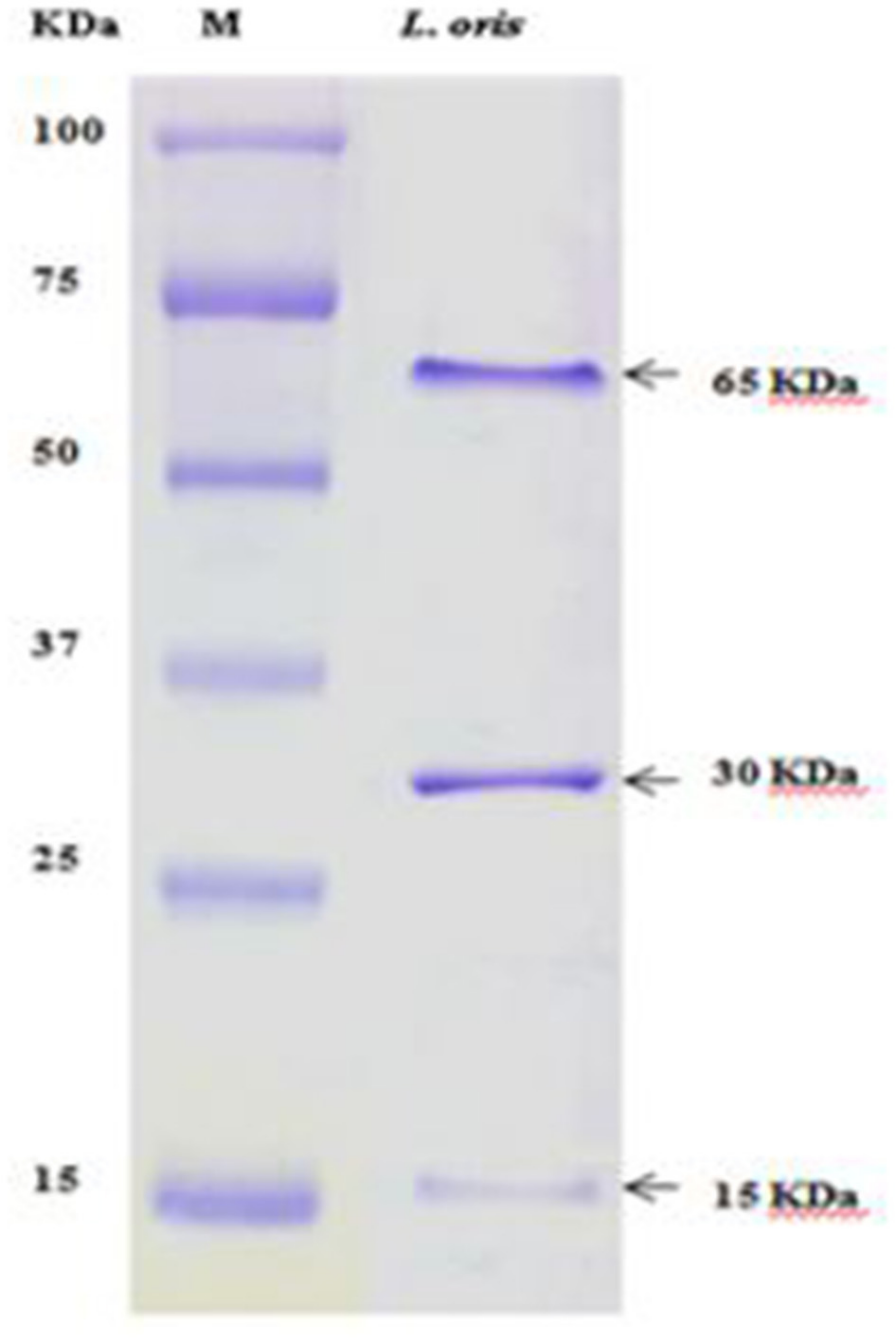
SDS-PAGE analysis. M: Standard protein marker.

The partially purified protein of *L. oris* was assessed through SDS-PAGE (10% gel) and evaluated with a standard protein marker (Figure 5). Analysis of the differentially accumulated proteins of *L. oris* demonstrated molecular weights of approximately 65, 30, and 15 kDa. The molecular weights of isolated proteins were compared with a protein ladder and also a standard protein solution (albumin-68 kDa, casein-24 kDa, and β-lactoglobulin-18 kDa).

### Cholesterol Reduction Assay

**Figure 6:**
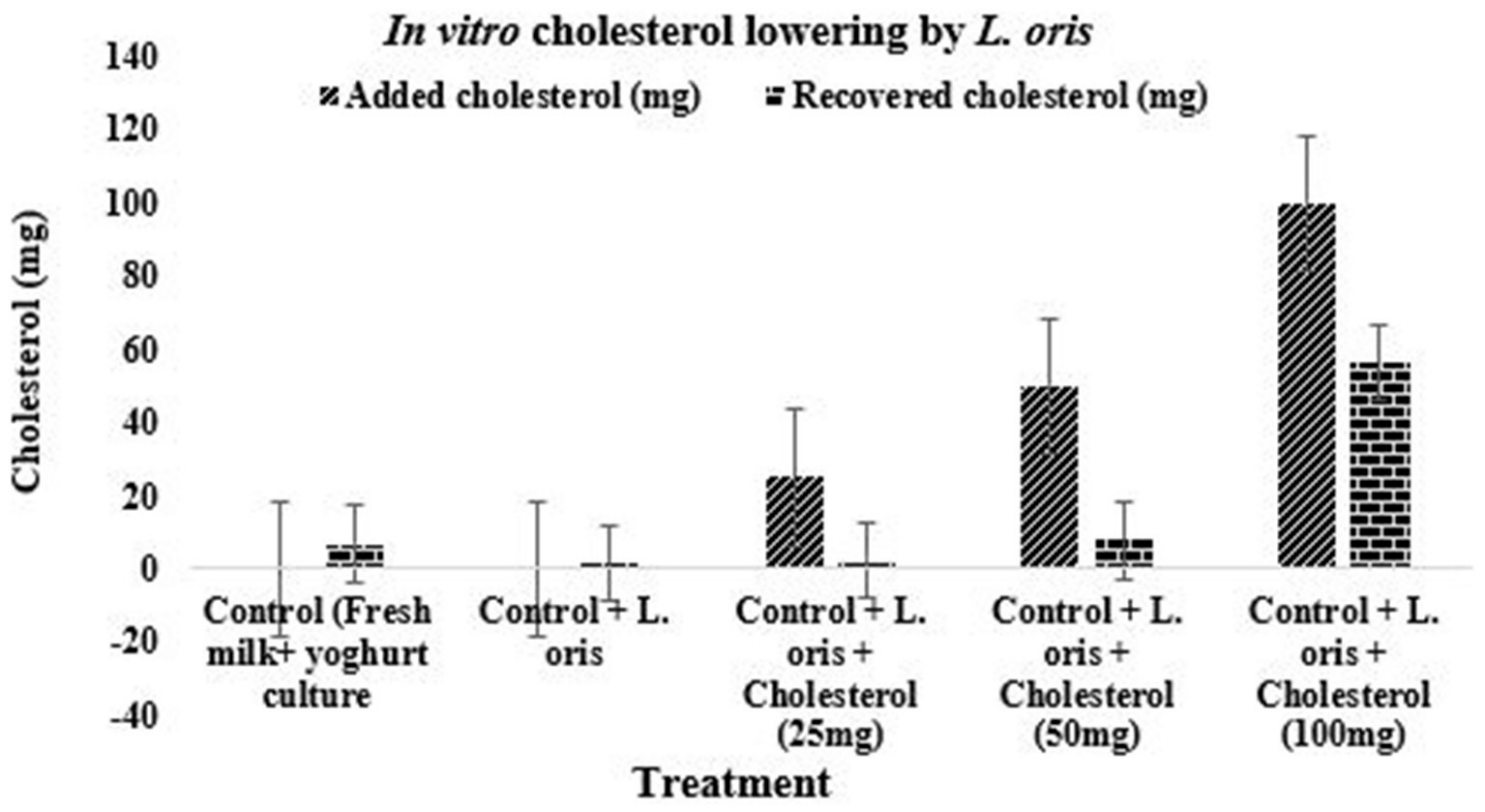
Cholesterol-lowering effect of *L. oris* strain.

Figure 6 indicated the cholesterol-lowering effect of the newly isolated probiotic strain *L. oris*. As a larger amount of cholesterol is the common physiological problem among people and the findings demonstrated very promising cholesterol-lowering activities. The probiotic *L. oris* can reduce cholesterol up to 90% depending on the concentration of cholesterol.

## Discussion

Probiotics are live microorganisms that have immense benefits for health. Until recently, a number of probiotic organisms were isolated from raw or processed milk, but in the case of mother's milk limited data have been documented. Breast milk is a vital factor for the development and composition of the neonatal gut microbiota aiming at assisting the suitable growth of the gut microbiota and the immune development of infants and an attractive source of new and specific probiotic strains. Earlier reports have indicated that the importance of milk microbiota is connected to the observation that the GI microbiota is drastically different between breastfed and non-breastfed infants (44, 45). These previous reports indicated that *Enterococcus* sp., *Streptococcus* sp., *Micrococcus* sp., and *Staphylococcus* sp. were commonly found in human breast milk. Martín *et al* reported *Lactobacilli* such as *L. gasseri, L. rhamnosus, L. acidophilus, L. plantarum*, and *L. fermentum* from mother's milk (10, 11). The results of this study indicated that a mother's milk can be used as a potential natural source for new and specific probiotic strains from *Lactobacillus* spp.

Among the seven potential *Lactobacillus* sp. isolates in this study, only C-1 was identified as *L. oris*. The identification of *Lactobacillus* to the species level was done by the BioLog^Tm^ identification system (22). The isolated *L. oris* strain was able to grow optimally at 37°C under anaerobic conditions (46) but unable to grow at 10°C. The strain was heat stable and able to grow at 45°C. Resistance to bile concentration and low pH are both characteristics of the identified isolate, and are considered to be vital properties of probiotics (46). To reach the small intestine, they have to pass through the stressful surroundings of the stomach. Although in the stomach, pH can be as low as 1.0, in most *in vitro* assays pH 3.0 has been preferred (46). The identified strain was resistant to acid at pH 3.5 for 90 min at 37°C. Also, the isolate was able to grow at pH 3.5 containing 0.2% mixed bile salts. The optimal salt tolerance capacities of *L. oris* were up to 2% and followed by growth rate declining. The bile salt tolerance test specified that the isolated *L. oris* probiotic was capable of surviving proficiently at up to 1.5% bile salt concentration at 2 h but survivability declined drastically after 5 h.

In a research study, 29 *Lactobacillus* strains of dairy origin were examined *in vitro* as prospective probiotics. All of the examined strains were resistant to pH 3 during 3 h, but most of them lost their viability in 1 h at pH 1.0. Also, all of them were tolerated by 0.3% bile salts concentration for 4 h (47). They were identified as *L. casei, L. plantarum, L. paracasei* subsp. (48). This study indicated that *L. oris* was able to grow and colonize in high bile concentration in the intestine, even at pH 1.0 in the stressful surroundings of the stomach. The isolate can retain a viability of approximately 80% at pH 1.0 after 5 h. A similar result was also found by Anandharaj et al. (16). Resistance to bile salt is one of the criteria used to select probiotic strains that would be potentially able to perform effectively in the human gastrointestinal tract (49). Gastric juice tolerance at pH 3.0 was examined. *L. oris* had better viability in gastric juice for 3 h at pH 3.0. After 3 h the viability was decreased. The viability of gastric juice at pH 6.0 was examined as a control. Additional reports designated that the growth rates of *L. gasseri, L. sakei*, and *L. acidophilus* decreased to 11–91% in simulated intestinal juices with 0.2% oxgall (50). The cell hydrophobic, autoaggregation and co-aggregation properties in the intestinal epithelial cells are considered to be probiotic criterion. The *L. oris* showed a good hydrophobic affinity and autoaggregation ability increases over time which matches the previous study result (30, 51). The co-aggregation ability of the isolated *L. oris* probiotic was acceptable in comparison to other fecal pathogenic and non-pathogenic microbes.

The production of antimicrobial compounds against pathogens is an imperative property of probiotics. *Lactobacillus* spp. contained antimicrobial substances that can inhibit the growth of pathogens. Darsanaki *et al* showed that *L. plantarum* (3 isolates), *L. casei* (2 isolates), and *L. brevis* (1 isolate) produced the highest tolerance against acid and bile salts as well as antimicrobial activities against *Staphylococcus aureus* PTCC 1431, *S. typhimurium* PTCC 1639 and *E. coli* PTCC 1399 (52). In the present study, *L. oris* rendered pronounced growth and inhibitory zones ranged between 7 and 8 mm against *S. aureus, S. enteritidis, V. parhaaemolyticus*, and *B. cereus*. Nevertheless, *L. oris* exhibited a consistent reduction in inhibitory zones against the other bacterial pathogens tested. Savadogo et al. (53), confirmed the antimicrobial activity of other *Lactobacillus* strains. *L. fermentum* formed the maximum inhibition zone (12 mm) against *Enterococcus faecalis* at the same time the minimal inhibition was shown by *Leuconostoc mesenteroides* subsp. *mesenteroides* on the same strain. In an additional study, *L. salivarius* FC113 created an inhibition zone of 11.5 ± 0.7 mm and 14.3 ± 0.6 mm against *S. aureus* strains KCCM 11335 and KCCM 11593, respectively (54). This inhibitory effect may be due to the production of organic acids and other antimicrobial substances (16).

*Lactobacillus* spp. can act as an antioxidant by quenching free oxygen radicals. These free radical scavenging properties of *L. oris* possibly indicate small amount of redox potential in the intestine. Synthetic antioxidants have serious carcinogenic health effects in terms of safety and long term utilization (55). Therefore, it is vital to explore non-toxic, natural, and low-cost antioxidants as a substitute for a synthetic antioxidant. In the present study, the cell-free extracts of *L. oris* exhibited strong antioxidant activity. Lactic acid bacteria have a diversity of mechanisms to tolerate oxygen and reactive oxygen species, and the mechanisms change among species. Some evidence confirmed that *L. casei, L. acidophilus*, and *L. plantarum* have been used as model probiotics (24, 37, 56). In a previous study, this *Lactobacillus* sp. exhibited strong resistance to hydrogen peroxide (35). Likely, the potential isolates *L. oris* can resist H_2_O_2_. Nitrite is usually used as a food additive and is widely found in a variety of food products. It is a vital N-nitrosamines precursor that actively causes cancer and other diseases. Moreover, nitrites also react with amines to form N-nitroso compounds (57). It is, therefore, an essential safety issue to limit the use of nitrite in foodstuff. In this circumstance, the tested isolate can deplete nitrite and restrict the conversion of nitrite into nitrosamines.

The isolated probiotic must be safe for human consumption as an important criterion for selecting an organism as a probiotic. For this purpose, the antibiotic resistance profile of the isolated organism was determined. The Agar well-diffusion method is used to detect the antimicrobial susceptibility of intestinal microorganisms. According to the European Food Safety Authority (EFSA), the assessment of hemolytic activity is mightily recommended if the isolated bacteria are intended for use in food, even if they have GRAS or QPS (Quality Presumption of Safety) status. Moreover, the organism *L. oris* also passed safety evaluation examination. In this study, the hemolytic activity of the tested isolate evaluated the strains *L. oris* and showed no hemolytic activity. The present results were similar to the findings of Oh and Jung who determined the hemolytic activity of five *Lactobacillus* species isolated from traditionally fermented beverages (58). The present findings were also consistent with the results of Wang et al., who estimated the probiotic potential of lactic acid bacteria from Chinese spontaneously fermented non-dairy food products, which revealed no hemolytic activity of probiotics (59).

The electrophoretic pattern of the proteins from the isolate showed a different band size. The partially purified proteins of *L. oris* on SDS-PAGE displayed molecular weights of approximately 65, 30, and 15 kDa have also been reported. The molecular weights of isolated enzymes were compared with a protein ladder and also a standard protein solution (albumin-68 kDa, casein-24 kDa, and β-lactoglobulin-18 kDa). Ghazi et al. (60) prepared a standard protein solution and compared it with *Lactobacillus* strains. The SDS-PAGE technique generated multifaceted and constant patterns that were easy to interpret and compare the profiles of the strains.

The *L. oris* strain was found to reduce cholesterol by 43.78 ± 6.97 to 90.50 ± 2.32% depending on the different concentrations of cholesterol in the *in vitro* test. The cholesterol assimilated by the *L. oris* showed wide variation within 6/7 h in anaerobic incubation. The amount of cholesterol decomposition or assimilation was higher in the low concentration of cholesterol followed by increments. The highest amount of cholesterol reduction was found in 25 mg/100 mL yogurt which was 90.50 ± 2.32% and followed by 84.60 ± 6.03%, and 43.78 ± 6.94% for 50 mg and 100 mg of cholesterol in the same amount, respectively. There was a significant difference (*P* < *0.05*) in the reduction of cholesterol in different concentrations. The recovered amounts of cholesterol in the control sample with yogurt culture were 6.80 ± 1.02 mg and the control sample with *L. oris* culture was 1.32 ± 0.73 mg. Some of the previously published results were directly agreed with the results of this study (16, 42, 61). The above results indicated that *L. oris* had a clear effect on *in vitro* cholesterol reduction which was dependent on *L. oris* counts and cholesterol amount. Development of various probiotic products, such as fermented milk, drinks, yogurt, cheese, ice-cream etc. with the help of *L. oris* culture, could confer health benefits for people in Bangladesh and other countries. Based on the results of the present study, *L. oris* has great probiotic properties and the ability to survive in human gastrointestinal conditions. *L. oris* needs to be further investigated and could be incorporated into food supplements or developed as a new probiotic.

## Conclusion

The use of probiotics as food or dietary supplement is increasing gradually with increasing awareness of its beneficial effects. In this study, one isolate was identified as *L. oris* and the experiment observation of probiotic capability revealed that the strain was a probiotic with excellent potential. The cell hydrophobicity, auto and co-aggregation properties in the intestinal epithelial cells were promising. *L. oris* indicates resistance to the majority of antibiotics as well as antagonistic activity against tested pathogen. Furthermore, the strain demonstrated promising cholesterol-lowering properties based on cholesterol level and its ability to survive in the gastrointestinal environment. These results reveal that *L. oris* has superior probiotic properties and can be regarded as a probiotic candidate, potentially opening new directions for future research into enhanced effectiveness and advancing biotechnological research in the food and dairy industries.

## Data Availability Statement

The original contributions generated for this study are included in the article/Supplementary Material, further inquiries can be directed to the corresponding author/s.

## Ethics Statement

The studies involving human participants were reviewed and approved by the BCSIR Institutional Ethical Review Committee. The participants or their legal guardian (sample donors) provided written consent to participate in this study.

## Author Contributions

MH designed the study. SAf and SAk executed the experiments. MH and SB provided advice and guidelines on conducting this research. MH, SAf, and SAk wrote the draft. All authors read and approved the final version of the manuscript.

## Conflict of Interest

The authors declare that the research was conducted in the absence of any commercial or financial relationships that could be construed as a potential conflict of interest.

## Supplementary Material

Supplementary Figure 1 Identification of *L. oris* by BioLog^TM^ Identification system.
