# Supplementary figures and images for: The Prospects of Lactobacillus oris as a Potential Probiotic With Cholesterol-Reducing Property From Mother's Milk
Source: Front Nutr. 2021 Mar 4;8:619506. doi: 10.3389/fnut.2021.619506 (PMC7969506; doi:10.3389/fnut.2021.619506)

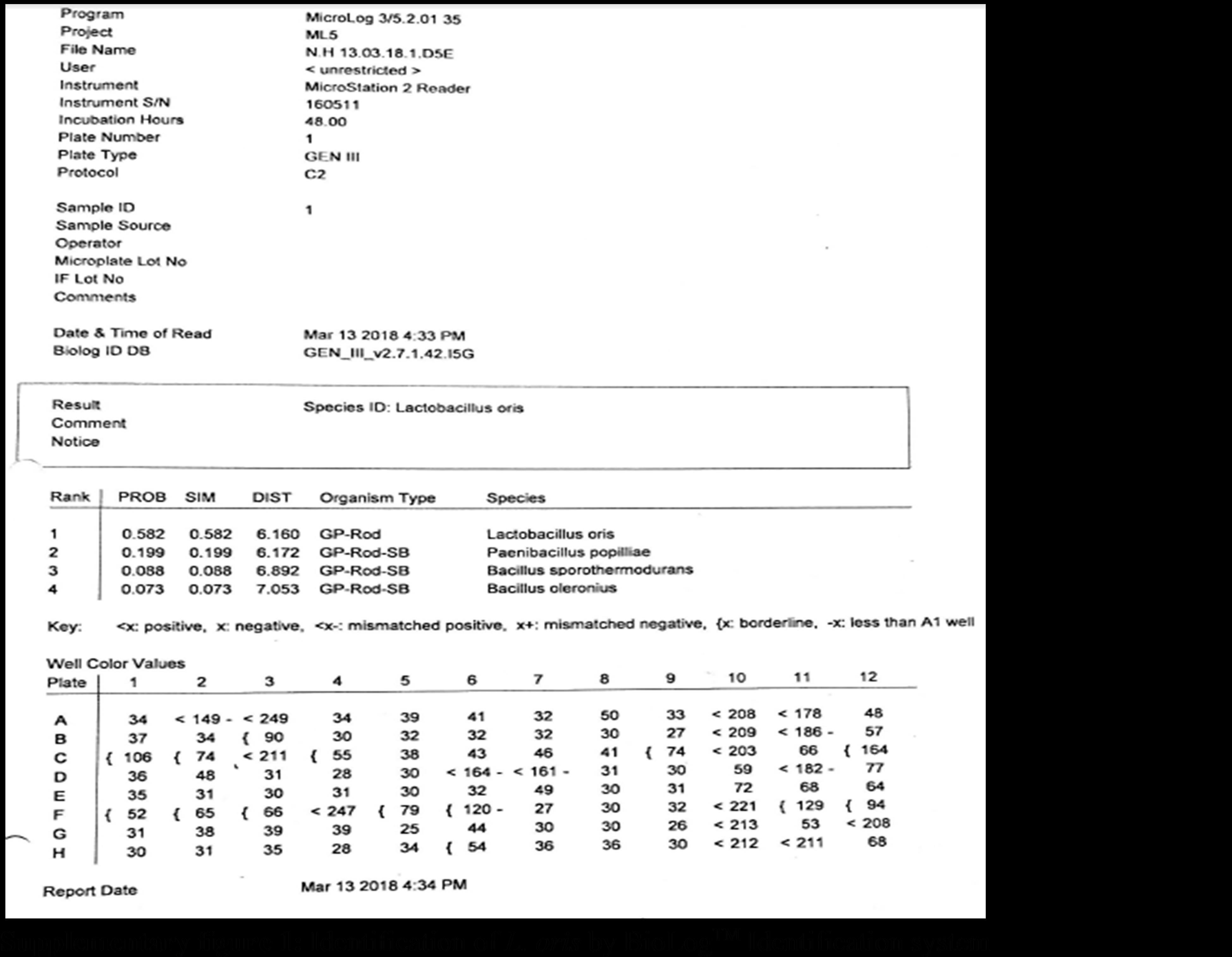

Supplement: Supplementary Figure 1 — Identification of L. oris by BioLogTM Identification system. [file Image_1.JPEG]
